# IKAP Deficiency in an FD Mouse Model and in Oligodendrocyte Precursor Cells Results in Downregulation of Genes Involved in Oligodendrocyte Differentiation and Myelin Formation

**DOI:** 10.1371/journal.pone.0094612

**Published:** 2014-04-23

**Authors:** David Cheishvili, Paula Dietrich, Channa Maayan, Aviel Even, Miguel Weil, Ioannis Dragatsis, Aharon Razin

**Affiliations:** 1 Familial Dysautonomia Centre, Pediatric Department Hadassah Hospital Hebrew University Hadassah Medical School, Jerusalem, Israel; 2 Department of Physiology, College of Medicine, The University of Tennessee, Health Science Center, Memphis, Tennessee, United States of America; 3 Laboratory for Neurodegenerative Diseases and Personalized Medicine, Department of Cell Research and Immunology, The George S. Wise Faculty of Life Sciences, The Sagol School of Neurosciences, Tel Aviv University, Ramat Aviv, Tel Aviv, Israel; 4 Department of Developmental Biology and Cancer Research, Institute of Medical Research Israel-Canada, The Hebrew University Hadassah Medical School, Jerusalem, Israel; The University of Tennessee Health Science Center, United States of America

## Abstract

The splice site mutation in the *IKBKAP* gene coding for IKAP protein leads to the tissue-specific skipping of exon 20, with concomitant reduction in IKAP protein production. This causes the neurodevelopmental, autosomal-recessive genetic disorder - Familial Dysautonomia (FD). The molecular hallmark of FD is the severe reduction of IKAP protein in the nervous system that is believed to be the main reason for the devastating symptoms of this disease. Our recent studies showed that in the brain of two FD patients, genes linked to oligodendrocyte differentiation and/or myelin formation are significantly downregulated, implicating IKAP in the process of myelination. However, due to the scarcity of FD patient tissues, these results awaited further validation in other models. Recently, two FD mouse models that faithfully recapitulate FD were generated, with two types of mutations resulting in severely low levels of IKAP expression. Here we demonstrate that IKAP deficiency in these FD mouse models affects a similar set of genes as in FD patients' brains. In addition, we identified two new IKAP target genes involved in oligodendrocyte cells differentiation and myelination, further underscoring the essential role of IKAP in this process. We also provide proof that IKAP expression is needed cell-autonomously for the regulation of expression of genes involved in myelin formation since knockdown of IKAP in the Oli-neu oligodendrocyte precursor cell line results in similar deficiencies. Further analyses of these two experimental models will compensate for the lack of human postmortem tissues and will advance our understanding of the role of IKAP in myelination and the disease pathology.

## Introduction

Familial Dysautonomia (FD) is a neurodevelopmental, neurodegenerative, autosomal-recessive genetic disorder primarily affecting the Ashkenazi Jewish population with an incidence of 1∶3,700 live births [1], [2]. FD patients show defective function and gradual degeneration of the autonomic nervous system as well as impairment of the sensory and motor nervous systems, which lead to an array of clinical manifestations, including ataxic gait, failure to thrive, impaired temperature and pain perception, and dysautonomic crisis [3]. Neuropathological findings include reduced numbers of neurons in sympathetic and sensory ganglia, reduced skin and target organ innervation, selective loss of unmyelinated and myelinated nerve fibers, and optic nerve atrophy [3], [4]. Although the central nervous system has been poorly investigated in FD, MRI neuroimaging suggests compromised myelination and white matter micro-structural integrity [5]. Sudden death due to autonomic instability, aspiration pneumonias, and respiratory insufficiency are the leading causes of death [3].

The most common mutation, which accounts for 99.5% of all FD patients, is a T to C transition in position 6 of the donor splice site of intron 20. This mutation causes skipping of exon 20 and results in expression of both wild type and mutant (missing exon 20) transcripts [6], [7]. It was demonstrated that this mutation reduces stable base pairing between the splicing factor U1 and the 5′ splice site of exon 20, shifting splicing of exon 20 from constitutive to alternative splicing [8]. Additionally, the major mutation alters *IKBKAP* gene splicing efficiency in a tissue-specific manner. All examined FD tissues and organs produce both transcripts, full size and exon 20 skipping, with the nervous system expressing significantly lower levels of full-length IKAP (the protein product of the *IKBKAP* gene) [6], [9].

There is much controversy in the literature concerning the function of the IKAP protein. The original identification of IKAP as an I-kB kinase (IKK) complex-associated scaffolding protein suggested that this protein functions in the formation of the IKK complex and in interaction with NF-kB [10]. However, subsequent analyses failed to show association of IKAP with IKKs [11], [12]. Later work supported the idea that IKAP is a component of the six-subunit complex of RNA polymerase II Elongator in humans [13], [14]. This complex is thought to have a role in transcriptional elongation, and altered transcription of several genes has indeed been shown in FD-derived fibroblasts as well as in HeLa cells transfected with IKAP siRNA [15]. However, IKAP is mostly a cytosolic protein. IKAP binds and induces JNK (cJun N-terminal kinase) activity, suggesting that it may be a physiologically important regulator of the stress-associated JNK signaling pathway [16]. IKAP was also shown to be involved in tRNA modification, exocytosis, cell adhesion, migration, and microtubule organization [17]–[22]. Although all these findings implicate IKAP in multiple processes, the underlying mechanisms leading to FD are still unknown.

In an attempt to elucidate the mechanisms underlying FD neuropathological and clinical features, we previously analyzed the RNA expression pattern from brains of two FD patients and two age-matched controls. Our analyses revealed that out of the top 25 genes that were significantly down-regulated (>2-fold) in both FD patient brains, thirteen of them are known to be involved in the process of oligodendrocyte differentiation and myelination [23]. The observed large number of genes involved in myelination - a function that in the brain is specifically carried out by mature oligodendrocytes - suggested that IKAP may play a pivotal role in this process. Despite the potential significance of these results, due to the scarcity of FD brain tissues, they were based on exploration of only two FD samples. Therefore it was especially important to validate these results in other FD models.

*IKBKAP* is highly conserved in evolution. The mouse IKAP protein shares ∼80% identity with the human orthologue and the consensus donor splice site of intron 20 that is mutated in the major FD haplotype is also conserved in the mouse *IKBKAP* gene [24], [25]. An FD mouse model therefore would have been highly justified and informative. However, despite the high level of homology, the creation of an FD mouse model recapitulating the FD major haplotype turned out to be a difficult task. A first attempt by Slaugenhaupt and colleagues, involving the introduction of the human *IKBKAP* locus with the FD major mutation into bacterial artificial chromosome and integrating it into the mouse genome was fruitless [26]. More recently, a knock-in humanized FD mouse model was generated in which human exon 20 and the two flanking introns containing the mutation of the major haplotype replaced the reciprocal mouse genomic sequences. However, this FD mouse model appeared to be fully viable and did not manifest any of the FD symptoms [27].

Inactivation of the *IKBKAP* gene in the mouse also does not recapitulate FD. Instead it leads to early embryonic lethality and developmental retardation associated with cardiovascular abnormalities and defects in telencephalon development [28], [29]. Embryos homozygous for an *IKBKAP* allele where exon 20 is deleted (*IKBKAP* Δ20) are indistinguishable from *IKBKAP* knockout embryos, indicating that deletion of exon 20 also leads to embryonic lethality [29].

Recently, the first mouse models for FD that display disease specific phenotypes were generated. Compound mutant mice carrying one *IKBKAP* allele in which exon 20 was deleted (*IKBKAP* Δ20 allele) and a hypomorphic allele that resulted from the insertion of loxP sites surrounding exon 20 (*IKBKAP* flox allele) recapitulate the severe end of FD spectrum, while in mice homozygous for the hypomorphic *IKBKAP* flox allele the FD characteristics are significantly milder [30]. Characterization of these models showed that they both recapitulate several FD features including severely reduced IKAP expression, increased perinatal lethality, low birth weight, failure to thrive, reduced growth rate, reduced number of fungiform papillae in the tongue, gastrointestinal dysfunction, ataxia, skeletal abnormalities, and impaired development and maintenance of sensory and autonomic nervous systems [30]. Although these mice recapitulate FD in many aspects, the CNS of these models has not been characterized.

Here we show that genes involved in oligodendrocyte maturation and myelin formation are also significantly downregulated in FD mouse brains, thus validating the results previously reported in FD patient brains. Knockdown of IKAP in the oligodendrocyte precursor cell line Oli-neu also recapitulates these deficits, indicating that IKAP has a cell-autonomous function in oligodendrocytes regulating the expression of genes involved in myelin formation. Our results provide further insights into FD mechanisms and also demonstrate for the first time the role of IKAP in myelination.

## Results

### IKAP deficiency in FD mouse models results in downregulation of myelin-related genes

**Figure 1:**
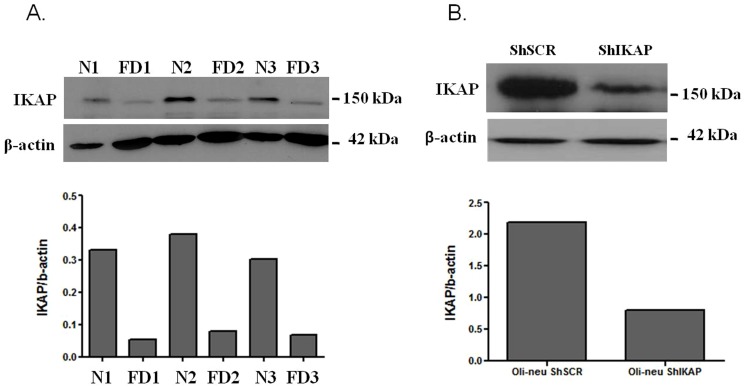
IKAP protein levels are highly reduced in FD mutant mice and after lentiviral ShIKAP administration in Oli-neu cells. (**A**) Western blot analyses of total protein lysates from brain cortex of control (N1, N2, N3) and age-matched FD (FD1, FD2, FD3) littermates. Upper panel shows detection of IKAP with the polyclonal anti-IKAP antibody (AnaSpec), and lower panel shows anti-β-actin for loading control. Note that IKAP protein expression is highly reduced in FD1, FD2, and FD3, relative to controls. Quantitative analysis of the Western blot was performed using Image J software. IKAP expression levels over β-actin levels are presented. (**B**) Western blot analyses of total protein lysates from control scrambled (ShSCR) and *IKAP* knockdown (ShIKAP) Oli-neu cells. Upper panel shows detection of IKAP with the polyclonal anti-IKAP antibody (AnaSpec), and lower panel shows anti-β-actin for loading control. Note that IKAP is significantly expressed in ShSCR Oli-neu cells. Quantitative analysis of the Western blot was performed using Image J software. IKAP expression levels over β-actin levels and are presented.

In our previous study we have shown significant downregulation of genes involved in oligodendrocyte differentiation and myelination in FD human brains [23]. However, due to the scarcity of FD brain tissues, our analysis was limited to two FD patients only, and thus required further validation. The availability of mouse models that recapitulate most of FD phenotypic and neuropathological findings provided us with an opportunity to validate our results [30]. These particular FD mouse models were generated by introducing mutations in the mouse Ikbkap gene that result in severely reduced levels of IKAP expression in the nervous system and recapitulate most of FD phenotypic and pathological features, including sensory and autonomic deficits [30]. Since the process of myelination in the central nervous system of mice is complete already at one month of age [30], FD mice older than one month of age and age-matched heterozygous or wild-type control littermates were used for our analyses (Table S1). Reduced expression of IKAP in the brains of FD mice was verified by Western analyses, and quantitative analyses confirmed that IKAP expression in FD mouse brains is significantly reduced, ranging from 10 to 20% that of control mice (Fig. 1A).

**Figure 2:**
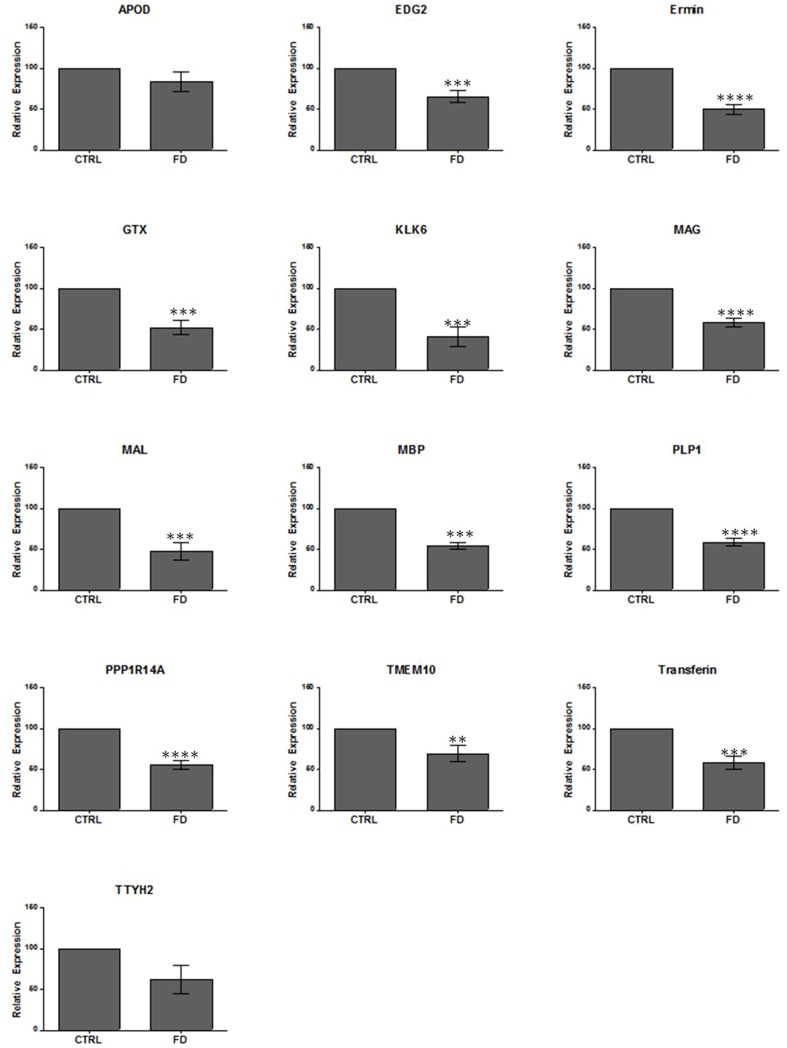
Reduced expression of IKAP target genes in FD mouse models. Quantitative real-time PCR (qPCR) analysis of APOD, EDG2, Ermin, GTX, KLK6, MAG, MAL, MBP, PLP1, PP1R14A, TMEM10, Transferrin, and TTYH2 in brain RNA from control (CTRL, n = 8) and FD (n = 7). Analysis was repeated 2 to 3 times for each gene. Expression levels were normalized over internal control (see Materials and Methods) and are expressed as percentage of CTRL. Data are represented as Mean ±SD. **P<0.01, ***P<0.001, ****P<0.0001.

**Figure 3:**
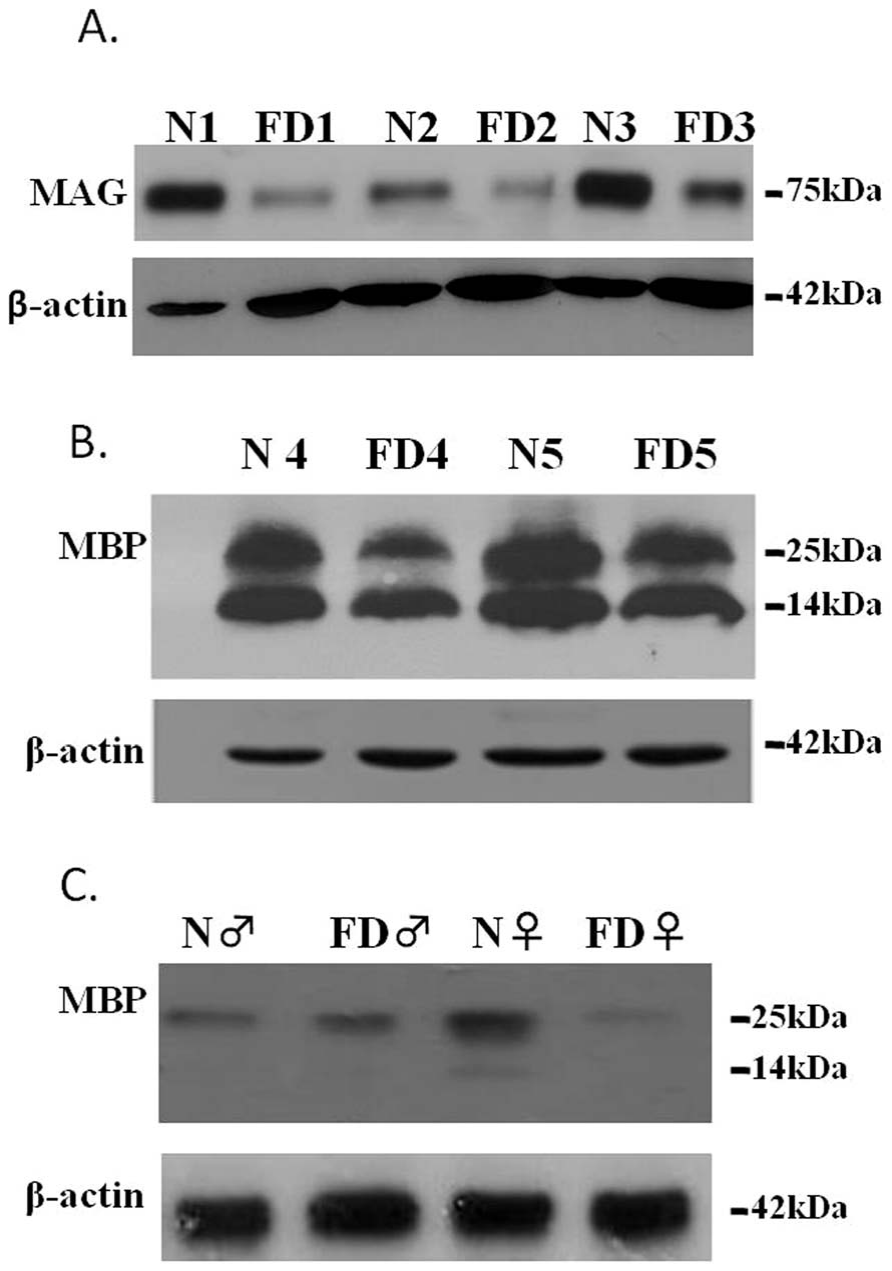
Reduced levels of MAG and MBP proteins in FD brains. (**A**) Western blot analyses of total protein lysates from brain cortex of control (N1, N2, N3) and age-matched FD (FD1, FD2, FD3) mice. Upper panel shows detection of MAG and lower panel shows anti-β-actin for loading control. Note that MAG protein expression is reduced in FD1, FD2, and FD3, relative to control brains. (**B**) Western blot analysis of total protein lysates from brain cortex of control (N4, N5) and FD (FD4, FD5) mice. Upper panel shows detection of MBP and lower panel shows β-actin for loading control. Note that there is reduction in the levels of both MBP isoforms (25 kDa and 14 kDa) in FD brains compared to controls. (**C**) Western blot analyses of total protein lysates from control male and female individuals (N) and FD male and female patients. Upper panel shows detection of MBP and lower panel shows -β-actin for loading control. Note the significant reduction in MBP expression in the female FD patient compared to its age-matched female control.

Since FD is caused by highly reduced IKAP levels in CNS of FD patients, and having confirmed the low level of expression of IKAP in our FD mutant mice, we have now in hand a suitable system to allow us to verify the involvement of IKAP in oligodendrocyte differentiation and myelin formation. To this end, we performed quantitative PCR (qPCR) in RNA derived from brain cortex of control (n = 8) and FD (n = 7) mice. As shown in Figure 2, similar to what we had observed in FD patients [23], we observed 30–60% reduction in expression of mRNAs encoding the myelin components proteolipid protein 1 (PLP1) and Myelin Associated Glycoprotein (MAG), myelin and lymphocyte protein (MAL), the late-stage oligodendrocyte-specific transcription factor GTX and cytoskeletal protein Ermin, as well as PPP1R14A, TTYH2, EDG2, APOD, Transferrin (TF), and TMEM10 whose expression is considerably induced in differentiating oligodendrocytes [31], [32]. These results were confirmed at the protein level using specific polyclonal antibody against MAG, by Western blot assay (Fig. 3A). Since expression of the RNA encoding the major myelin structural protein PLP1 and the minor myelin component MAG were both significantly downregulated in FD brains, we further screened for additional target genes known to be involved in myelin formation. We found that expression of the myelin basic protein (MBP), the second major protein component of myelin, was also significantly reduced in FD mouse brains at both the RNA (Fig. 2) and protein levels (Fig. 3B). Similarly, expression of kallikrein-related peptidase 6 (KLK6), involved in myelination and myelin turnover [33], was also significantly downregulated in FD mouse brains (Fig. 2).

Downregulation of MBP expression in FD mouse models prompted us to examine its level of expression in FD patients' brains. We performed Western blot analysis (Fig. 3C) using protein extracted from the brain of an 11-year-old FD male patient and a 47-year-old female FD patient and of sex and age matched unaffected individuals reported previously [23]. Interestingly, we observed a significant reduction of MBP protein in the 47-year old FD patient's brain compared to a normal age and sex counterpart, while downregulation in an 11-year-old FD male compared to its normal counterpart was not significant. This apparent difference between the two FD patients could potentially be explained by the fact that in humans the process of myelination continues through adolescence, up to 20 yeas of age [31].

### IKAP deficiency in mouse oligodendrocyte precursor cell line results in downregulated expression of genes involved in the myelination process

In the central nervous system, the process of myelination is specifically carried out by mature differentiated oligodendrocytes. Oligodendrocyte maturation occurs stepwise, and the precision of timing and sequential order by which myelination occurs in the mammalian CNS indicates that highly localized signaling mechanisms derived from astrocytes and/or neurons must also participate in the regulation of oligodendrocyte differentiation and myelination [34], [35].

**Figure 4:**
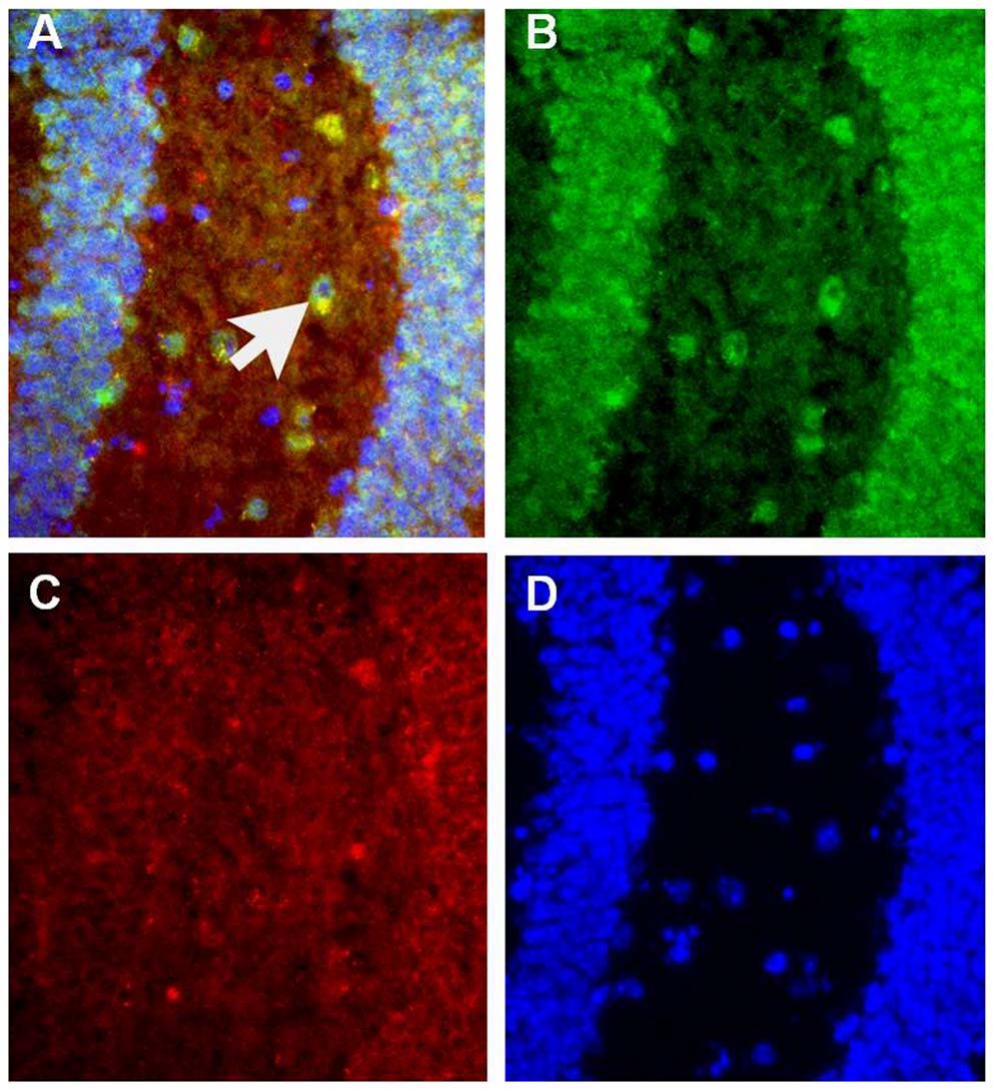
IKAP is expressed in oligodendrocytes in vivo. Sagittal mouse brain cryosection at the level of dentate gyrus immunostained for IKAP (green) and the oligodendrocyte marker GALC (red), counterstained with DAPI (blue). (**A**) Arrow shows an oligodendrocyte, which stains for GALC and IKAP simultaneously (yellow). (**B**) IKAP expression (green channel), (**C**) GALC expression (red channel), and (**D**) DAPI staining (blue channel).

In vitro analyses however indicate that differentiation of oligodendrocytes from the precursor stage to the mature cell is identical in culture as in intact tissue, even without the presence of neurons. Thus oligodendrocyte progenitors have the intrinsic capacity to differentiate into mature oligodendrocytes without the need of cell-to-cell contact. Therefore the downregulation of myelin-related genes observed in FD brains might reflect either an environmental signaling deficit, or alternatively IKAP may be required cell-autonomously in oligodendrocytes for the regulation of expression of genes involved in myelination. Immunohistochemistry for IKAP and the oligodendrocyte-specific marker galactosylceramidase (GALC) in mouse brain indeed confirmed that IKAP is expressed in oligodendrocytes in vivo (Fig. 4). To address if IKAP is required cell-autonomously, we used a lentiviral Sh-RNA system to knockdown IKAP expression in the Oli-neu mouse oligodendrocyte precursor cell line [36] (see Materials and Methods). The Oli-neu cell line has been extensively characterized and used as a reliable cellular model to study oligodendrocyte differentiation [32], [37]. Moreover, recent functional genome analyses indicates that this line is already farther along in process of differentiation compared to immature precursor cells, since it expresses significantly higher levels of several mRNAs such as PLP1 and MBP, among others [38], making it suitable for our proposed analyses.

**Figure 5:**
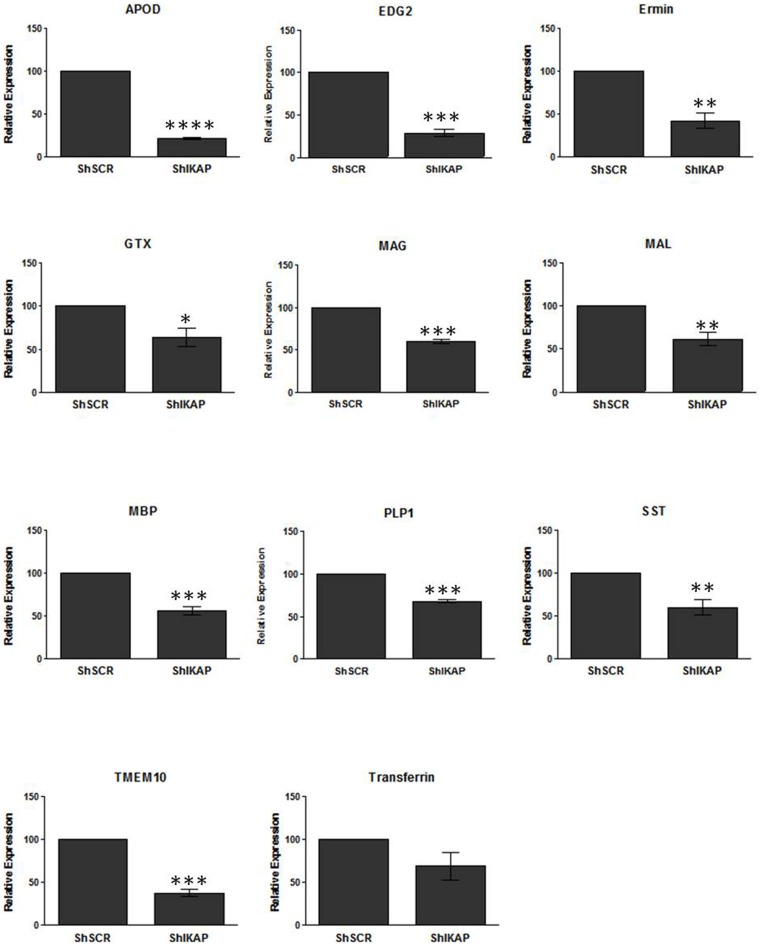
IKAP knockdown in Oli-neu cells results in reduced expression of myelin-related genes. Quantitative real-time PCR (qPCR) analysis of APOD, EDG2, Ermin, GTX, KLK6, MAG, MAL, MBP, PLP1, PP1R14A, SST, TMEM10, Transferrin, and TTYH2 in RNA from control (ShSCR) and Ikbkap knockdown (ShIKAP) Oli-neu cells. Analysis was repeated 2 to 3 times for each gene. Expression levels were normalized over internal control (see Materials and Methods) and are expressed as percentage of CTRL. Data are represented as Mean ±SD. *P<0.05, **P<0.01, ***P<0.001, ****P<0.0001.

As detailed in Materials and Methods, Oli-neu cells were transfected with lentiviral Sh-RNA, treated with puromycin for selection, and RNA and protein extracts were prepared one week later. Expression of IKAP in native Oli-neu cells and its downregulation following lentiviral IKAP Sh-RNA was confirmed by qPCR and Western blot analysis (Fig. 1B, Figure S1). We then performed qPCR analysis for genes associated with oligodendrocyte differentiation and myelination. The mature oligodendrocyte-specific genes Ermin, GTX, MAL, MAG, and MBP were all downregulated about 50% in IKAP Sh-RNA cells compared to control cells (Fig. 5), i.e. levels that are similar to those observed in FD mouse brains (see Fig. 2). These results indicate that IKAP is required cell-autonomously in oligodendrocytes for ensuring high levels of expression of myelin-related genes. Significantly, we found that although expression of APOD and EDG2 is downregulated four-fold in IKAP Sh-RNA Oli-neu cells compared to controls (Fig. 5), their downregulation is considerably smaller (30–40% decrease) in FD mouse brains. The apparent discrepancy can potentially be explained by the fact that both EDG2 and APOD are not expressed only in oligodendrocytes. Notably, although APOD and EDG2 expression is highly induced in mature oligodendrocytes, expression of APOD is also strongly induced in glia (astrocytes and microglia) upon brain injury or neurodegeneration [39], while EDG2 is upregulated in response to ischemia [40].

In summary, our results indicate that IKAP has a cell-autonomous major role in the regulation of expression of myelin-related genes in oligodendrocytes.

## Discussion

Despite the implication of IKAP in multiple cellular processes [21], the mechanisms underlying FD clinical and neuropathological findings are still poorly understood. In an attempt to elucidate the mechanisms underlying FD neuropathological and clinical features, we had previously conducted microarray expression analyses using RNA derived from brains of two FD patients and two age-matched controls. Our analyses revealed that out of the top 25 genes that were significantly downregulated in both FD patient brains, thirteen of them are known to be involved in the process of oligodendrocyte differentiation and myelination, suggesting a pivotal role for IKAP in this process [23]. Despite the potential significance of these results, we relied on only two FD and two normal individuals, and therefore these results required further validation.

In the current manuscript we have taken advantage of recently generated FD mouse models [30] to validate our findings. Creating an appropriate animal model for a genetic disorder is one of the most important approaches that allows studying not only the function of the mutated gene, but also enables the researcher to further understand the disease process and screen for potential drugs. The FD models used in this study were previously shown to recapitulate several FD features including severely reduced IKAP expression, increased perinatal lethality, low birth weight, reduced growth rate, reduced number of fungiform papillae in the tongue, ataxia, skeletal abnormalities, and impaired development and maintenance of sensory and autonomic nervous systems [30]. Our analyses presented herein demonstrate that these models also recapitulate the oligodendrocyte-specific molecular changes observed in FD patients' brains, underscoring their suitability for furthering our understanding of FD. We also provide proof that IKAP expression is needed cell-autonomously for the regulation of expression of genes involved in myelin formation since knockdown of IKAP in the Oli-neu oligodendrocyte precursor cell line results in similar deficiencies.

High-speed conduction and fidelity of signaling over long distances, are some of the major advantages conferred to the vertebrate nervous system by the myelin sheath. The importance of myelin in human development is highlighted by its involvement in a number of different neurological diseases such as leukodystrophies and multiple sclerosis in the CNS and peripheral neuropathies in the PNS [31].

In FD patients, neuroimaging findings revealed abnormalities in white matter consistent with compromised myelination [5], loss of dorsal column myelinated axons in the spinal cord [41], [42], while sural nerve biopsies revealed absence of thin myelinated fibers as well as large caliber myelinated fibers, while medium myelinated fibers display abnormal morphology [43]–[45]. Our findings that IKAP expression is required for proper expression of genes related to myelination in CNS oligodendrocytes suggest that IKAP may also play a similar role in myelinating Schwann cells of the peripheral nervous system and can potentially explain some of FD clinical and pathological findings. The expression of IKAP protein in Schwann cells in DRGs of chick E10 in ex-ovo explants (Miguel Weil, unpublished results) further supports this hypothesis.

The two major structural proteins of myelin are PLP and MBP. PLP constitutes about 50% by weight of myelin proteins [46]. PLP leads to increased myelin stability; in the absence of PLP, myelin is unstable and slowly degenerates [47]. MBP constitute 30–40% of myelin proteins by weight, and is also required for myelin stabilization [46], [48]. MAG, composing less than 1% of myelin proteins is important in the development of myelin sheath. More specifically, MAG is the major mediator of axonal-glial contacts that are essential for the initiation of myelination [49]. Our findings indicate that in FD brains mature oligodendrocytes express reduced levels of genes that encode the major myelin proteins MBP, PLP1, and the minor myelin protein MAG (Fig. 2 and 3). Since the formation of large caliber fibers is strongly dependent on the amount of myelin produced by its associated myelinating cells and stability of myelin requires proper amounts of its structural components [47], [48], reduced myelination and/or myelin instability can potentially underlie the absence of large caliber fibers, and potentially also loss of functional muscle spindle function and the ataxic gait of FD patients [50]. In addition, defects in myelination or myelin stability can also contribute to progressive optic nerve atrophy [51] a frequent occurrence in FD patients [52]; and abnormal deposition of myelin around axons can also lead to decreased nerve conduction velocities [53] a finding observed in the sural nerve of FD patients [54].

Although myelination is an essential process in the central and peripheral nervous system little is known about the mechanisms of myelination or the signals that regulate this complex process. Our findings implicate IKAP as one of the major players in the process of myelination. IKAP could be required for regulation of myelin genes either by its direct involvement in mRNA transcription elongation, as demonstrated for several genes in fibroblasts [15] or by regulating expression of upstream transcription factors. In support of the latter hypothesis, in FD brains as well as in IKAP Sh-RNA Oli-neu cells, expression levels of the homeobox transcription factor GTX are significantly reduced (Fig. 2 and 5). Significantly, binding sites for GTX are present in the promoter regions of MBP and PLP and mobility shift assays confirmed that GTX indeed binds to these promoter regions [55], and increases in GTX expression parallels that of myelin genes in differentiating oligodendrocytes as well as in the re-myelination process [55], [56]. While other transcription factors are likely to be required for regulation of expression of myelin genes [57], our finding that IKAP is required for GTX expression suggests that reduced expression of myelin genes in IKAP-deficient cells might be a secondary consequence.

In brief our results indicate that IKAP is required cell-autonomously for maintenance of high levels of expression of myelin-related genes and that abnormal myelination may contribute to FD pathology and clinical manifestations. Further analyses of these two experimental models will compensate for the lack of human postmortem tissues and will advance our understanding of the role of IKAP in myelination and the disease pathology.

## Materials and Methods

### Generation of mice and genotyping

This study was carried out in strict accordance with the recommendations in the Guide for the Care and Use of Laboratory Animals of the National Institutes of Health. The protocol was approved by the Animal Care and Use Committee of the University of Tennessee Health Science Center.

Mice were housed and bred at the University of Tennessee Health Science Center Comparative Medicine Department animal core facility. The details of generation of the mice and their characterization has been presented elsewhere [30]. Mice were interbred, and the progeny was genotyped by PCR using genomic DNA isolated from tail biopsies, as described [30].

### Tissue harvest

Mice for tissue harvest were subjected to euthanasia by CO2 inhalation, followed by cervical dislocation. Whole brain from the FD mouse model and control mice were dissected out and quick-frozen at -80°C (Table S1).

FD and normal human brain tissues were obtained from the NICHD Brain and Tissue.

Bank for Developmental Disorders at the University of Maryland, Baltimore, MA. Tissues used for this study were the cerebrum of an FD 11-year-old patient (UMB #M3697M) and a 47-year-old FD female (UMB #M3783M) and age and sex matched normal brain (UMB# 616 and UMB# 1910, respectively) [23].

### Analysis of brain sections by Immunofluorescence

Sagittal brain cryosections (12 µm) of 6 months old mice were fixed using 4% PFA for 10 minutes, washed several times with PBS and incubated with blocking solution (8% horse serum, 1% BSA, 0.3% Triton-X and 0.02% Sodium Azide) for 1 hr at room temperature. Primary antibodies specific for IKAP (Rabbit anti hIKAP, H-302, Santa Cruz biotechnologies) at dilution of 1∶70 and anti Galc Mab (a gift from Dr. Ari Barzilai laboratory, Tel Aviv University) at dilution of 1∶50 in blocking solution were applied onto sections overnight at 4°C. Sections were washed 3 times with PBS and then incubated with secondary Antibodies (Alexa Fluor 488 Anti-Rabbit, Alexa Fluor 594 Anti-Mouse from Life technologies, USA) for 1 hr at room temperature. Finally sections were washed and stained with DAPI in anti fading mounting solution (Vecta Shield, Vector Laboratories Inc.) then visualized and photographed using a Nikon Eclipse 80i fluorescent microscope attached to a Nikon DS-Fi1 CCD camera.

### Cell culture

Oli-neu cells [36], a gift from J. Trotter, University of Mainz, were grown on poly-L-lysine-coated culture dishes in Sato medium consisting of DMEM supplemented with 2 mg/ml NaHCO3, 2 mM L-glutamine, 10 µg/ml Apo-Transferine, 10 µg/ml Insulin, 100 µM Putrescine, 200 nM Progesterone, 500 nM TIT, 220 nM Sodium Selenite, 520 mM L-Thyroxin, 1% Horse serum, Gentamicin.

293T human embryo kidney cells were grown in Dulbecco modified Eagle medium (DMEM) in the presence of 10% fetal bovine serum (FBS) and antibiotics. Cells were maintained at 37°C in 5% CO_2_ in a humid atmosphere.

### RNA Interference Treatment

To suppress *IKBKAP* gene expression, lentiviral (pLKO) vector was used. Five different double stranded *IKBKAP* shRNA, accession number NM_026079 were purchased from Sigma-Aldrich (Israel).

The day before transfection, 1.2×10^6^ HEK293T cells were plated in 10 cm (20–30% confluence). The next day, the lentivirus was generated by co-transfection of 3 vectors (PLkO, transfer vector – includes the insert (Sigma), PHR – Lentivirus polymerase, VSVG – virus envelope) into 293T cell, performed by FuGene (Roche) according to the manufacturer's protocol. Three vectors were transfected at the following ratio PHR:VSVG:LVTR1 = 9∶1∶10. Cells were incubated for 48 hours followed by collection of the medium containing the virus and passed through a 0.45 um filter.

5×10^5^ Oli-neu cells were plated per well in 6 well plates the day before infection. The medium was discarded from the relevant cells and 1 ml of infection medium and 1 ml of relevant regular medium o/n were added. Next, the cells were washed with PBS and regular fresh medium was added. For selection, puromycin at a concentration of 1 µg/ml was added after 48 hours incubation. Cells were harvested 7 days after infection for RNA or protein extraction.

### Western immunoblotting

Cells and brain tissue were lysed in RIPA buffer (150 mM NaCl, 0.1% sodium dodecyl sulfate (SDS), 0.5% sodium deoxycholate, 50 mM Tris HCl, 1% NP-40, 1 mM phenylmethylsulfonyl fluoride). The protein concentration of the soluble fraction was measured by the Bradford assay. Total cell lysates were resolved by 8% SDS-polyacrylamide gel electrophoresis and transferred to a nitrocellulose membrane. After staining the membranes with reversible Ponceau red solution to confirm equal protein loading, the membranes were blocked with 5% Skim Milk in PBS (0.1% Tween-20) for 1 hour at room temperature. The membranes were then subsequently incubated in PBS with 0.1% Tween20 at room temperature for 1 h with the relevant primary antibodies. After three washes in PBS 0.1% Tween-20, the blots were incubated with appropriate secondary antibodies (final antibody concentration was according to the manufacturer's recommendation). Polyclonal antibody to IKAP was purchased from Anaspec (Cat# 54494), and for MAG was purchased from Santa Cruz Co., (Cat# SC-15324). Anti-MBP rabbit polyclonal IgG was purchased from Millipore (Cat# AB980). Monoclonal antibody to β-actin was purchased from Sigma Aldrich Co. (Cat#A5441). All secondary antibodies were purchased from Jackson Laboratories.

### RNA extraction and qPCR analysis

Total RNA was prepared from mouse brain and Oli-neu cell line using the Tri-reagent (Sigma) according to the manufacturer's protocol. Approximately 1 µg total RNA of each sample was reverse transcribed using the M-MLV Reverse Transcriptase (Promega) and random primers in a 20 µl reaction mixture. The reverse transcription reactions were carried out at 42°C for 30 min. For each gene, the real-time PCR analysis was performed in triplicates with 2 µl of the cDNA, primers (Table S2) at a concentration of 0.3 µM. The SYBR Green (Applied Biosystems) was added to the 20 µl reaction mixture. An ABI PRISM 5700 Sequence Detection System (Applied Biosystems) was programmed as follows: one cycle of 10 min at 95°C, followed by 40 cycles of 15 s at 95°C and 1 min at 60°C. Data were analyzed using the 7500 SDS system software. To control for the amount of RNA, amplification of TBP was performed and corrected for each cDNA sample.

Relative expression results are presented as changes in the threshold cycle (ΔΔCt). The threshold cycle refers to the PCR cycle at which the extent of fluorescence is increased to a calculated level above background. Statistical analysis was undertaken using Prism (GraphPad Software, Inc). The significance was set at P<0.05.

### Statistical analysis

Statistical analysis for qPCR data was done using GraphPad Prisma Software (Moher et al., 2009). Each value represents the mean ±SD of 2 or 3 independent experiments. The results were considered statistically significant when P<0.05.

## Supporting Information

Figure S1 Expression profile of IKAP targeted genes in healthy control mouse (left) and in ShSCR Oli-neu cells. Relative expression of IKAP target genes was measured by qPCR. Relative expression is calculated as Log10.
(TIF)

Table S1 Genotype and age of control (N) and FD mice used for analyses.
(PDF)

Table S2 Primers used for qPCR analyses.
(PDF)
