# Supplementary figures and images for: IKAP Deficiency in an FD Mouse Model and in Oligodendrocyte Precursor Cells Results in Downregulation of Genes Involved in Oligodendrocyte Differentiation and Myelin Formation
Source: PLoS One. 2014 Apr 23;9(4):e94612. doi: 10.1371/journal.pone.0094612 (PMC3997429; doi:10.1371/journal.pone.0094612)

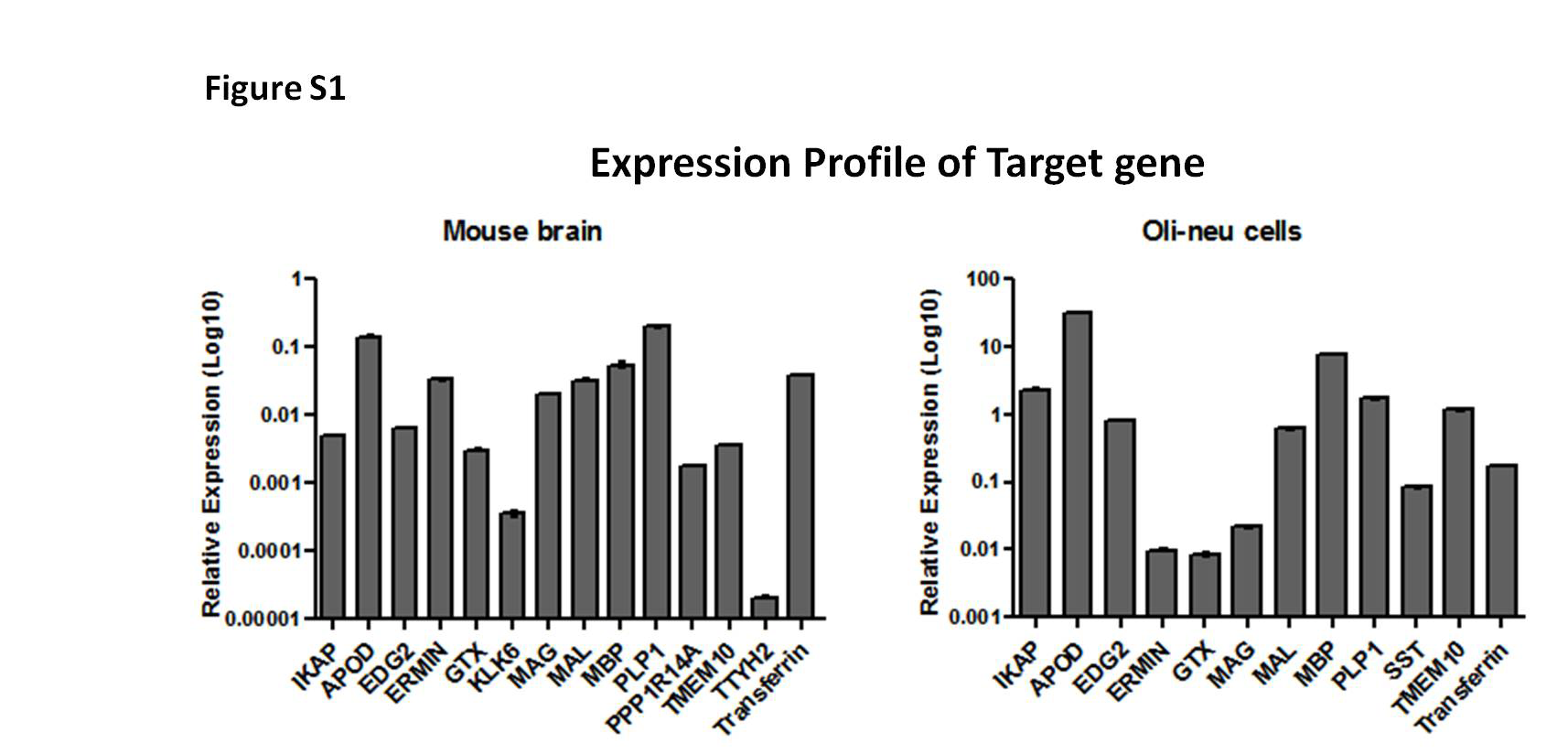

Supplement: Figure S1 — Expression profile of IKAP targeted genes in healthy control mouse (left) and in ShSCR Oli-neu cells. Relative expression of IKAP target genes was measured by qPCR. Relative expression is calculated as Log10. (TIF) [file pone.0094612.s001.tif]
