# Supplementary material for: IKAP Deficiency in an FD Mouse Model and in Oligodendrocyte Precursor Cells Results in Downregulation of Genes Involved in Oligodendrocyte Differentiation and Myelin Formation
Source: PLoS One. 2014 Apr 23;9(4):e94612. doi: 10.1371/journal.pone.0094612 (PMC3997429; doi:10.1371/journal.pone.0094612)
Supplement: Table S1 — Genotype and age of control (N) and FD mice used for analyses. (PDF) [file pone.0094612.s002.pdf]

Table S1

Genotype and age of control (N) and FD mice used for analyses

Sample Name	Genotype	Age
N1	*Ikbkap*$^{+/+}$	1 month
FD1	*Ikbkap*$^{\Delta 20/flox}$	1 month
N2	*Ikbkap*$^{flox/+}$	3 months
FD2	*Ikbkap*$^{flox/flox}$	3 months
N3	*Ikbkap*$^{+/+}$	11 months
FD3	*Ikbkap*$^{flox/flox}$	11 months
N4	*Ikbkap*$^{flox/+}$	20 months
FD4	*Ikbkap*$^{flox/flox}$	20 months
N5	*Ikbkap*$^{flox/+}$	16 months
FD5	*Ikbkap*$^{flox/flox}$	16 months
N6	*Ikbkap*$^{flox/+}$	16 months
FD6	*Ikbkap*$^{flox/flox}$	16 months
N7	*Ikbkap*$^{flox/+}$	19 months
FD7	*Ikbkap*$^{flox/flox}$	19 months
N8	*Ikbkap*$^{+/+}$	11 months

Please note that since the process of myelination in the central nervous system of mice is complete already at one month of age, FD mice older than one month of age and age-matched heterozygous or wild-type control littermates were used for our analyses.
