# Supplementary material for: IKAP Deficiency in an FD Mouse Model and in Oligodendrocyte Precursor Cells Results in Downregulation of Genes Involved in Oligodendrocyte Differentiation and Myelin Formation
Source: PLoS One. 2014 Apr 23;9(4):e94612. doi: 10.1371/journal.pone.0094612 (PMC3997429; doi:10.1371/journal.pone.0094612)
Supplement: Table S2 — Primers used for qPCR analyses. (PDF) [file pone.0094612.s003.pdf]

Table S2

Primers used for qPCR

Genes	Primer sequences for QPCR
**IKAP**	For: 5' TGTTTCTCGTCTCCCGTGTG 3'<br>Rev: 5' AGCAATTTCTACGACGCCCT 3'
**PLP1**	For: 5' GGCTAGGACATCCCGACAAG 3'<br>Rev: 5' GGCAAACACCAGGAGCCATAC 3'
**Transferrin**	For: 5' TGCATCAAGGCCATTTCTGC 3'<br>Rev: 5' CCGGCATCGTACACCCAAC 3'
**GTX**	For: 5' GAAGGTGTGGTTCCAGAATCG 3'<br>Rev: 5' TTTTAGCCGACGCCATCTCT 3'
**PPP1R14A**	For: 5' GGAAAGATGCCGGAAAATCC 3'<br>Rev: 5' GCTCCTGGACGAAGTCCTCTG 3'
**MAG**	For: 5' TGCTTGCCTAGCAGAGAACG 3'<br>Rev: 5' AAGGTGCATACATGACACTCAGCT 3'
**MAL**	For: 5' TCACACTGGATGCAGCCTACC 3'<br>Rev: 5' CAGGGCTTCCAGAACTGAGG 3'
**TTYH2**	For: 5' TGACCTGGTCATCTGCCTTG3'<br>Rev: 5' GCATGGAGGCTAGGAGACACTT 3'
**MBP**	For: 5' ACATTGTGACACCTCGAACACC 3'<br>Rev: 5' GCCAAATCCTGGCTTCTGC 3'
**EDG2**	For: 5' CCAGGAGGAATCGGGACAC 3'<br>Rev: 5' GACAATAAAGGCACCAAGCACA 3'
**APOD**	For: 5' GGTGAAGCCAAACAGAGCAAC 3'<br>Rev: 5' GGTGGCATCAACGGGAAG 3'
**Ermin**	For: 5' TCACCAGGCCATCAAAGATCT 3'<br>Rev: 5' GATGCCCTTCTCGGAAAGC 3'
**KLK6**	For: 5' CCTGGCAAGATCACCCAGAG 3'<br>Rev: 5' TCACCCTGACAGGAATCGTTG 3'
**TMEM10**	For: 5' GCTGGTGGCTTTGCTGTTTAC 3'<br>Rev: 5' CAGTCTCCTCTACGGGCTCATC 3'
**SST**	For: 5' GGAAACAGGAACTGGCCAAG 3'<br>Rev: 5' GGCATCATTCTCTGTCTGGTTG 3'
